# Data from the Swiss TREE Panel Study (Transitions from Education to Employment)

**DOI:** 10.5334/jopd.97

**Published:** 2023-11-09

**Authors:** Sandra Hupka-Brunner, Thomas Meyer, Andrés Gomensoro

**Affiliations:** 1 University of Bern, Switzerland; 2 Universities of Bern and Geneva, Switzerland

**Keywords:** School-to-work transition, life course research, educational & occupational trajectories, vocational education and training (VET), Switzerland

## Abstract

TREE (Transitions from Education to Employment) is a prospective inter-disciplinary mixed-mode panel study following up on post-compulsory education and employment trajectories of two Swiss compulsory school leavers’ cohorts: TREE1 was launched as a PISA follow-up survey in 2000 (n_t0_ = 6343, n_t10_ in 2020 = 3882). TREE2 started in 2016 and draws on a national large-scale assessment of mathematics skills (n_t0_ = 8429, n_t6_ in 2022 = 4461). The panel is ongoing, further panel waves being planned for both cohorts. Data from both cohorts are available at the Swiss data archive SWISSUbase (www.swissubase.ch; reference number 12476).

## (1) Background

### Research objectives and theoretical background

TREE’s objective is to observe long-term trajectories and transitions within and between the (post-compulsory) education and the labour market systems in Switzerland, with particular focus on the individual development of young people during these phases.

Analysing long-term trajectories of multiple cohorts calls for a theoretical framing that best addresses the specificities of different biographical phases as well as contextual changes at the macro and meso levels that may affect the observed cohorts. Furthermore, the multi-disciplinary character of TREE requires a framework that relates to the relevant discourses in the involved research fields. Therefore, it must be open to further development and adaptation of the survey programme – be it due to biographical and life-cycle developments, social changes between cohorts, or methodological and theoretical innovations in the respective research fields.

As a general theoretical framework, the life course research paradigm as initially proposed by Elder (1975) addresses these needs. In recent years, the paradigm has gained importance in a number of disciplines of the social sciences, thus lending itself to an overarching frame for inter-disciplinary use of the TREE data (Bernardi et al., 2019). It draws on both sociological (Elder, 1975) and psychological (Baltes et al., 2007) research traditions that are considered to be relevant for the transitions under scrutiny.^1^ As Diewald & Mayer (2009) emphazise both traditions assume that the embeddedness of individual developmental processes in historical and local contexts is crucial (e.g., age effects, big events such as COVID or societal trends as digitisation). In addition, there is widespread agreement that an individual’s life course and development are embedded in social contexts (significant others, institutions), and that transitions are developmental tasks (Havighurst, 1972 [1948]) in the face of which the individual must make decisions. Understanding individual agency therefore has to account for different life contexts in which adolescents develop and with which adolescents and their families must deal with (Bernardi et al., 2019) as well as for the timing of different events and stages within the life course. Furthermore, individual action is seen as guided by bounded rationality (Bernardi et al., 2019, Esser, 2004; Stocké et al., 2011).

While life course sociology sees the individual life course as strongly influenced by opportunity structures, institutional settings, and decision-making situations, life course psychology focuses primarily on intra-individual development and the individual’s adaptation to external conditions (Bernardi et al., 2019). With regard to the concepts and scales adopted in the TREE surveys, we refer to sociological theories of status reproduction on the one hand (Bourdieu, 1977; Bowles & Gintis, 2002; for a critical overview of the current state of research, see also Draelants & Ballatore; Winkler-Wagner, 2010) and rational choice approaches (Baumert & Schümer, 2002; Blossfeld & Shavit, 1993; Boudon, 1974; Breen & Goldthorpe, 1997) on the other. These theories model the mechanisms by which social origin influences educational decisions at each point of transition in the education system.

In the fields of psychology and educational sciences, TREE draws on developmental theories that deal with educational and occupational socialisation (Heinz, 1984; Ulich, 1991) as well as with the ways in which youths cope with, in Bronfenbrenner’s (1981) terms, “ecological transitions”. The underlying assumption is that individuals need certain skills to successfully manage transitions, such as the ability to set and pursue goals. Motivational aspects (Deci & Ryan, 1993; Wild, 2001), self-efficacy as well as problem-solving and decision-making skills are held to be the central characteristics that favour successful educational and acquisition pathways (Schoon & Silbereisen, 2009; Silbereisen et al., 2006). Eccles (2006) and Gottfredson (1981) underline that not only skills in their own right, but also their intra-individual representation (Greve, 2000) are crucial for educational choices and success. Other concepts used to explain educational pathways derive from theories of institutional resources and constraints (which may lead to individual stress; Semmer, 1997), wellbeing (e.g., self-esteem, depression, attitude towards life, personal values; see Hascher, 2004) and critical life events (e.g., relocation, divorce or separation of parents, illness, death of a relative; see Filipp, 1995).

When it comes to analysing labour market entry, TREE relies on concepts and instruments used by classical human capital theory (Becker, 1964), signal(ling) and social capital theories (Coleman, 1988; Spence, 1973), theories of discrimination (Arrow, 1994; Becker, 1957/1971) and school-to-work transition models based on labour market economics (OECD, 2000; Ryan, 2001; Ryan, 2004).

### The Swiss education System as a specific context of school-to-work transitions

The Swiss education system is characterized by a federal, small-knit structure and a pronounced horizontal and vertical stratification from lower secondary level onwards. Furthermore, there are historically and culturally determined differences between cantons and language regions which reflect varying institutional structures of opportunity (Glauser & Becker, 2016; for a detailed institutional self-description, see www.edk.ch).

In most cantons, after grade 6 of primary school (at around 12 years of age), students are streamed to two up to four different tracks of lower secondary school, which differ according to their level of academic requirements. The extent and form of tracking varies greatly from canton to canton. Approximately 30% of all students are assigned to tracks officially termed to fulfil “basic (academic) requirements”, with inter-cantonal variation ranging from 10 to 40 percent. Even though educational policy postulates permeability between tracks, initial allocation to a given track remains de facto largely irreversible (Bayard, 2018; Oesch, 2017). The tracking not only influences further skills development (Angelone & Ramseier, 2012; Baumert et al., 2006; Tomasik et al., 2018) but also further education trajectories (Gomensoro & Meyer, 2021; Scharenberg et al., 2016) and has proven to be a major factor reinforcing social inequality (Terrin & Triventi, 2022). Compulsory schooling ends after three years of schooling at lower secondary level (i.e., after nine school years).^2^

Upper secondary education in Switzerland is dominated by firm-based vocational education and training (VET): the large majority of Swiss compulsory school leavers (65 to 70%) attends VET programmes, which are provided in over 200 training professions, with varying duration (two and four years) and academic requirements. Most programmes are provided in a “dual” form, i.e., learners attend a (usually larger) part of their training “on the job” in a training company, while the remaining time is spent in (vocational) school. VET trainees in this type of programme sign an apprenticeship contract with the training firm and receive a (modest) salary. Vocational school attendance varies greatly by training profession, number of lessons and type of curriculum (for more detail, see e.g., Gronning & Kriesi, 2022). Upon completion of their training, they obtain a VET diploma which grants access to specific occupational segments of the labour market and/or to further training at post-secondary and tertiary levels. In particular, VET graduates may acquire a professional baccalaureate, which entitles them to access Universities of applied sciences.

Only about one third of all students enrolled in upper secondary level education attend academically oriented general education programmes.

Completion rates at tertiary level education is relatively low by international standards, particularly in the VET sector (for a general outline regarding VET and higher education, see Nikolai & Ebner, 2011).

## (2) Methods

### 2.1 Study design

**Figure 1:**
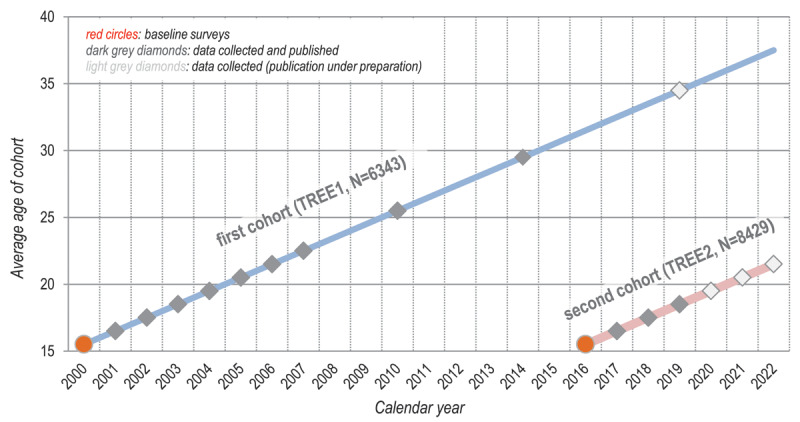
TREE multi-cohort design.

TREE (Transitions from Education to Employment) is a multi-disciplinary longitudinal large-scale survey on educational and occupational pathways in Switzerland for the use within the scientific community at large. The source of the data are two panel surveys of school leavers that both start at the end of compulsory school, at respondents’ age of approximately 15 to 16 years (see Figure 1), drawing on national large-scale assessments as baseline surveys (see Section 2.4.3 for detail); for further documentation, see the two cohorts’ detailed study designs (Gomensoro & Meyer, 2017; Hupka-Brunner et al., 2023; TREE, 2016d).

Since the launch of the first TREE cohort in 2000, the macro context in which educational pathways and transitions are embedded has changed. This includes major reforms to the education system that are likely to affect the educational pathways of young people. Moreover, global developments in the domains of economy, communications technology, environment, science and demographics pose major challenges to education systems worldwide (Blossfeld et al., 2007). The Swiss education system is no exception and is confronted with challenges such as the ongoing economisation and pluralisation of society (changing family forms, changing fertility birth patterns, increased international and national migration) and the increasing computerisation and virtualisation of everyday life (Akademien der Wissenschaften Schweiz, 2009; Hupka-Brunner et al., 2022).

Therefore, a second cohort has been launched in 2016 to examine the extent to which these changes affect the individual trajectories of youth. TREE2 adopts essential characteristics of the first cohort’s (TREE1) design, which allows for inter-cohort comparison of how school-to-work transitions have changed over time (Gomensoro & Meyer, 2017; TREE, 2016d). The two cohorts are comparable insofar as

– they both are followed up on their pathways through upper secondary and tertiary education into gainful employment and young to middle adulthood;
– they both are followed up at yearly intervals up to age 22–23, and at looser intervals (2–5 years) later on, thus providing a dense and comprehensive observation of all relevant types of activities during school-to work transitions;
– they both draw on a baseline survey including elaborate assessments of cognitive skills acquired by the end of lower secondary education;
– the baseline surveys of both cohorts provide comprehensive measurements of students’ starting conditions held to be relevant for their later educational and labour market pathways;
– they both draw on a large, nationally and regionally representative sample of students in their last year of compulsory education (i.e., at the end of lower secondary education).
– Moreover, response for both baseline surveys is extraordinarily high (PISA 2000: 95%; AES 2016: 93%; see BFS & EDK, 2002; Verner & Helbling, 2019 for details), which substantially facilitates measures to correct for non-response bias due to panel attrition.

The two cohorts differ primarily in the granularity of the observed trajectories, which is finer for the second cohort in the early stages of the panel, as well as a number of topical foci that seemed more relevant at the time of the 2^nd^ cohort’s launch (e.g., digitisation; for an overview of the two surveys’ topical foci and their comparability, see also Table 5).

### 2.2 Time of data collection

The first TREE cohort (TREE1) was launched in 2000 and draws on a large national (compulsory) school leavers’ sample (n = 6343) tested and surveyed on the occasion of Switzerland’s then first-time participation in PISA. Since then, the sample has been followed up by means of ten panel waves, the most recent one conducted in 2019/20. Data were collected during the last three to four months of the Swiss school year (March through June/July) to ensure comparability of educational situations across panel waves. As of panel wave 8, data collection started somewhat later in some waves and was extended to a longer period (i.e., March/April to September/October), which is justifiable as pathways at that stage are substantially less closely structured by the rhythm of the school year. Further panel waves are planned at five-years intervals. To date, TREE1 respondents have reached an average age of approximately 40 and been surveyed for a period of over 20 years, spanning from early adolescence up to early middle-age. The study thus has gradually grown into a full-blown life course survey.

The second TREE panel (TREE2) covers a comparable population of school leavers who completed compulsory education in 2016 (n = 8429). Since then, the second cohort has been surveyed six times at yearly intervals. Data collection was conducted in the same period of year as with the first cohort. Further panel waves are planned at looser intervals (two to five years).

### 2.3 Location of data collection

With respect to the first TREE cohort (TREE1) the baseline survey (PISA 2000) was administered in a proctored classroom setting within the schools drawn for the test sample. As to the subsequent TREE1 panel waves, there were no instructions or restrictions as to where or in which setting respondents were to complete the survey. It may be assumed that most respondents completed the questionnaires (both CATI and self-administered) at their homes. In later panel waves (and in step with the increasing use of cellular telephones and smartphones in the past decade), we have to assume a wide variety of locations where the survey was completed (at home, in school, at work, while commuting, etc.).

With regard to the second TREE cohort (TREE2), the administration of the baseline survey also took place in a proctored classroom setting within the schools drawn for the test sample. As smartphone use had become largely commonplace by the time of TREE2’s launch (2016), we have to assume – much like in the later panel waves of the first cohort — a wide variety of locations where the survey was completed (at home, in school, at work, while commuting, etc.).

### 2.4 Sampling, sample and data collection

#### 2.4.1 Sampling and sample of TREE’s first cohort (TREE1)

The TREE1 population comprises students who had attended a regular public school at lower-secondary level at the time of the PISA 2000 survey and completed compulsory education by the end of the school year 1999/2000. The sample is representative of Switzerland as a whole, the Swiss language regions (German, French, and Italian-speaking Switzerland) and selected cantons (Bern, Geneva, Ticino, St. Gall).

The Swiss PISA 2000 sample was designed to be representative of both ninth-graders and, regardless of their grade at the time of the PISA survey, fifteen-year-olds. The sampling adopted a two-stage, multiple disproportionate random selection with predetermined sample sizes for the two groups mentioned above, for language regions and for specific cantons (for details, see Renaud et al., 2000; Sacchi, 2011a, 2011b). Additionally and independently, a class sample was drawn from all ninth-grade classes in French-speaking Switzerland. The TREE study population is essentially identical to the Swiss PISA 2000 subsample of ninth-grade students. Given that PISA 2000 and TREE were designed as two distinctly separate surveys, PISA respondents were asked their consent to being contacted by TREE at a later stage and provide their contact details to this end. Approximately half of the initial PISA 2000 sample ended up doing so, were first surveyed by TREE in spring 2001 (panel wave 1) and henceforth at annual intervals up to 2007 (see Table 1; for further sampling details, see Sacchi, 2011a; Sacchi, 2011b).

**Table 1:** TREE1 sample and response.


Calendar year		2000	2001	2002	2003	2004	2005	2006	2007	2010	2014	2019/20

*Panel wave*		baseline	1	2	3	4	5	6	7	8	9	10

Ø *age of sample*		16	17	18	19	20	21	22	23	26	30	35

*Sample and response*	N valid gross sample		6343	5944	5609	5345	5060	4852	4659	4571	4402	3882

	N response		5528	5206	4877	4679	4506	4133	3979	3423	3142	2979

	% response/baseline		87%	82%	77%	74%	71%	65%	63%	54%	50%	47%


Table 2 provides an overview of the TREE1 sample by its main socio-demographic characteristics in 2000 (baseline survey) and 2014 (panel wave 9). The table highlights that in panel wave 9 (2014) and at an average cohort age of approximately 30, sample size still allows for analysis of a wide range of subgroups with regard to education, occupation, family and other situations. In order to correct for attrition bias, weights are provided.

**Table 2:** Selected sample descriptives for TREE1, baseline and panel wave 9 (2000 and 2014).


TREE1 BASELINE SURVEY, 2000		N	% OR MEAN

		(UNWEIGHTED)	(UNWEIGHTED)

**Total**		6343	100%

**Gender**	Female	3440	54%

	Male	2903	46%

**Age**		6319	Ø 15.5

**Year of birth**	1983	757	12%

	1984	3399	54%

	1985	2052	32%

	Other years	111	2%

**Academic track attended at lower secondary level**	Extended requirements	4358	68%

	Basic requirements	1626	26%

	No formal tracking	357	6%

**PISA reading literacy score**		6337	Ø 510.0

**Highest parental socio-economic index (HISEI**)		5845	Ø 50

**Highest parental education**	Compulsory Secondary Tertiary	1464 2189 2399	24% 36% 40%

**Migration background**	Swiss native*	3776	60%

	2^nd^ generation migrant**	1560	25%

	1^st^ generation migrant***	900	14%

**Language region**	German	2970	47%

	French	2540	40%

	Italian	833	13%

**Rural vs. urban area**	Rural	2072	33%

	Urban	4271	67%

**TREE1 PANEL WAVE 9, 2014 (≈30 YEARS OLD)**		**N**	**% OR MEAN**

		**(UNWEIGHTED)**	**(UNWEIGHTED)**

**Total**		3142	100%

**Educational attainment**	Compulsory	87	3%

	Secondary	1161	37%

	Tertiary	1881	60%

**Gainful occupation**	Full-time work (≥90%)	2074	66%

	Part-time work (<90%)	712	23%

	Other situation	356	11%

**Marital status**	Single	2202	70%

	Married	893	28%

	Other status	46	1%

**Parenthood**	Yes	767	24%

	No	2374	76%


Note that percentages may not add up to 100% due to missing values (not displayed for reasons of table readability). Note that we display only a (selected number of salient) variables measured/collected on the occasion of the two panels waves in question. * Participants and their parents born in Switzerland. ** Participants born in Switzerland, with at least one parent born abroad. *** Participants born abroad. Source: own calculations.

#### 2.4.2 Sampling and sample of TREE’s second cohort (TREE2)

The baseline survey of the second TREE cohort (TREE2), AES 2016, drew on a large, complex random sample of 22423 ninth-grade students. The sample was drawn by means of a two-step, disproportionally stratified sampling procedure with schools as primary sampling units. In cantons with small student populations, all students were drawn. Stratification aimed at obtaining sufficient sample sizes for analyses at cantonal level, which leads to a marked over-representation of small rural cantons. Moreover, students enrolled in tracks with low academic requirements were privileged in the drawing of some cantonal samples. For a detailed description of the complex sampling design we refer to Verner und Helbling (2019). After completion of the baseline survey, a sample of 13728 ninth-grade students had provided their contact details and their consent to being contacted by TREE at a later date. Due to restricted funding, TREE was not able to include all respondents who provided their contact details. In a first step, we therefore excluded most of the consenting respondents with incomplete baseline data. In a second step, we excluded another 2235 respondents by means of a randomized subsampling, leaving us with a gross initial panel sample of 9741 students.

The subsampling aimed to optimise sample composition in a longitudinal perspective. The general idea was to privilege respondent groups of particular analytic value and/or groups known to be particularly affected by panel attrition. Privileged inclusion of these groups was achieved by either omitting them from the subsampling altogether (i.e., including them in the sample with a probability of one) or by including them at an increased sampling probability (Hupka-Brunner et al., 2023; Sacchi, 2023).

The population covered by the TREE2 panel is almost identical to that of AES: AES basically includes all ninth-grade students enrolled in the school year 2015/16. For survey-practical reasons, about three per-cent of the students had been excluded from the AES (mostly students from schools for special needs; see Verner & Helbling, 2019). Furthermore, students who had repeated their 9th grade in the school year 2016/17 were excluded. By limiting the TREE2 population to (compulsory) school leavers, comparability of populations between the two TREE cohorts is maximised.

Table 3 highlights the evolution of TREE2’s response over the first six years of the panel. Compared to the response figures of TREE1 shown in Table 1, wave-specific response rates in TREE are distinctly lower, thus leading to a significantly higher degree of attrition in the younger cohort.

**Table 3:** TREE2 sample and responses.


*Calendar year*	2016	2017	2018	2019	2020	2021	2022

*Panel wave*	baseline	1	2	3	4	5	6

Ø *age of sample*	16	17	18	19	20	21	22

*Sample and response*							

N valid goss sample		9741*	9251*	8918*	7855	6949	6128

N response		7971	6903	6154	5353	4501	4461

% response/baseline		82%	75%	69%	68%	65%	73%


* Due to retrospective exclusions from the sample, the published datasets comprise only 8,429 cases.

Table 4 provides an overview of the TREE2 sample at baseline by its main socio-demographic features in 2016 (baseline survey) and descriptives of the educational status as of panel wave 3.

**Table 4:** Selected sample descriptives for TREE2, 2016 and 2019 (baseline and panel wave 3).


TREE2 BASELINE SURVEY, 2016		N	% OR MEAN

		(UNWEIGHTED)	(UNWEIGHTED)

**Total**		8429	100%

**Gender**	Female	4592	54%

	Male	3837	46%

**Age**		8429	Ø 15.8

**Year of birth**	1999	1038	12%

	2000	4809	57%

	2001	2492	30%

	Other years	90	1%

**Academic track attended at lower secondary level**	High requirements	2419	29%

	Extended requirements	3423	41%

	Basic requirements	2540	30%

**AES math score**		8340	Ø.13

**Highest parental socio-economic index (HISEI)**		8267	Ø 51

**Highest parental education**	Compulsory	1150	14%

	Secondary	3914	46%

	Tertiary	3174	39%

**Migration background**	Swiss native*	6093	73%

	2^nd^ generation migrant**	1541	18%

	1^st^ generation migrant***	763	9%

**Language region**	German	5885	70%

	French	2081	25%

	Italian	463	5%

**Rural vs. urban area**	Rural	4533	54%

	Intermediate	2001	24%

	Urban	1863	22%

**TREE2 3RD PANEL WAVE SAMPLE, 2019 (≈19 YEARS OLD)**		**N**	**% OR MEAN**

		**(UNWEIGHTED)**	**(UNWEIGHTED)**

**Total**		6154	100%

**Educational status**	Not in education or training	320	5%

	Internship or transitional solutions	92	1%

	2 years VET	73	1%

	3–4 years VET	2938	48%

	Vocational baccalaureate	612	10%

	General baccalaureate	1721	28%

	Other general education programme	398	6%


Note that percentages may not add up to 100% due to missing values (not displayed for reasons of table readability). Note that we display only a (selected number of salient) variables measured/collected on the occasion of the two panels waves in question. * Participants and their parents born in Switzerland. ** Participants born in Switzerland with at least one parent born abroad. *** Participants born abroad. Source: own calculations.

**Table 5:** Survey topics of TREE1 and TREE2.


SURVEY TOPICS			COMPARABILITY WITH TREE1

MAIN	DETAILED		

**Socio-demographics**	Socio-demographic characteristics and housing situation		

		Age and Gender	C

		Civil Status	C

		Housing situation	C

	Composition of (own) family		C

	Migration background and nationality		

		Migration background	C

		Nationality, residence status	

**Education, training and employment**	Educational pathways and transitions (lower sec. level)		

		Educational biography (compulsory school)	C

		Educational decisions (transitions lower => upper sec. education):perceived cost, benefit and chances of success	

		Educational objectives and aspirations	C

		Plans for education and training	C

		Characteristics of maths lessons (end of lower secondary education)	

	Educational situation and post-compulsory pathways		

		Attended educational programmes	C

		Attended schools	C

		Attended training firms	C

		Skills requirements for educational activities/media use	

		Absenteeism/intention to change education	C

		Resources and strains (education)	C

		Credentials and marks	C

		Reasons discontinuing education and training	

	Employment situation (incl. internships) and pathways		

		Employment/internships	C

		Conditions of employment	C

		Job position within company’s hierarchy	C

		Salary	C

		Resources and strains (employment)	C

		Job tasks, requirements and job-skills-mismatch	C

		Absenteeism/intention to change job	C

		Reasons for termination of employment	

		Desired job situation	

	Self-assessment of education and employment pathways		

		Assessment of current education and training	

		Assessment of completed education & training	

		Perceived fit and commitment: main activity	C

**Other activities, job and training search**	Search for education or employment		

		Search for education (end of lower secondary education)	

		Search for VET training place (upper sec.)	C

		Job search (upper sec.)	C

		Search for general education programme (upper sec.)	

	Other activities		

		Unemployment (unregistered and registered)	(C)

		Vacation/holidays	(C)

		Military service	(C)

		Childcare (as main activity)	(C)

		Illness/accident	(C)

		Maternity/paternity leave	(C)

		Gap/missing information	(C)

	Reasons for non-participation in education and employment		

		Reasons for non-participation in education and employment	

		Reasons for non-participation in education	

		Reasons for part-time & non-employment	

**Family, significant others, social origin and networks**	Family background		

		Family climate	C

		Socio-economic origin	C

		Childcare situation (own children)	

	Social, cultural, and economic resources		

		Social capital (own)	

		Cultural capital (family of origin)	C

		Cultural capital (own)	C

		Economic capital (family of origin)	C

		Financial situation (general)	C

**Social participation**	Social and cultural participation		

		Politics	(C)

		Leisure	

		Group affiliation and sense of belonging (identity)	C

**Well-beingand health**	Satisfaction and well-being		

		Satisfaction	C

		School-related well-being	

		Critical life events	C

	Health		C

**Self**	Non-cognitive factors		

		Motivational concepts	C

		Self-perception	C

		Emotions related to maths classes	

		Volitional strategies	C

		Personality characteristics	

		Global preferences (risk, time and social preferences)	

		Values and attitudes	C

		Attitudes related to maths classes	

	Cognitive skills (assessments)		

		basic mathematical skills	(C+)

		reading speed	

		cognitive skills	


*Legend for columns on comparison with TREE1:* C = Data (partly) comparable across cohorts. (C) Comparable data for both cohorts in upcoming data releases. (C+) Elaborated, but not fully comparable assessment of math competences available for both cohorts (TREE1: randomized split-half sample).

#### 2.4.3 Data Collection

As regards the first cohort TREE1, data for the baseline survey were collected in the context of Switzerland’s first participation in PISA, the OECD’s large-scale assessment of 15 year-olds’ literacy. PISA 2000 was conducted by means of the proctored administration of a paper-and-pencil questionnaire in a classroom setting. The survey consisted of a comprehensive literacy test^3^ and a student context questionnaire. It was conducted according to the standards of the OECD-led international PISA consortium (for more detail, see Renaud et al., 2000; Sacchi, 2011a, 2011b).

Data for TREE1 panel waves 1 to 4 were collected by means of a self-administrated paper-and-pencil questionnaire sent out to the participants’ home. The secondary mode was a proctored completion of the same questionnaire that was conducted by trained interviewers on the telephone.^4^

As of panel wave 5 (2005), data collection was conducted according to the general survey design adopted to this day for both TREE cohorts, i.e., a two-part questionnaire with an overall survey burden of approximately one hour. The main focus of the first part (base questionnaire BQ) is on the seamless capture (to the month) of all relevant education, employment and other activities (episodes or spells). The main survey mode of this part of the questionnaire is CATI (computer-assisted telephone interview), relying on reactive dependent interviewing,^5^ with a written, self-administered paper-and-pencil questionnaire as secondary mode in case of CATI non-response.^6^

After completion of the CATI, respondents receive an individually tailored complementary questionnaire (CQ) that collects in-depth data on their activities ongoing at the time of the survey (e.g., quality and conditions of their education or employment as well as various scales of self-assessment). Field work and data collection are mandated to a professional survey institute and conducted in close cooperation with the TREE survey management (for detail, see Gomensoro & Meyer, 2017; TREE, 2016a, 2016b, 2016c, 2016d, 2016e, 2016f, 2019).

The baseline survey of TREE2 is the Assessment of the Attainment of Educational Standards (AES), a large-scale assessment scheme for Swiss students in compulsory school carried out under the responsibility of the Swiss Conference of Cantonal Ministers of Education (EDK).^7^ The AES 2016 was conducted in the last year of respondents’ compulsory schooling (at approx. age 15) and administered as a classroom-based proctored computer-assisted self-interview (CASI), comprising a standardised mathematics test and a student context questionnaire.

In analogy to the TREE1 survey design outlined above, the TREE2 follow-up waves consist of two survey parts, the base questionnaire (BQ) and the complementary questionnaire (CQ). The primary mode of the BQ is a proactive, dependent computer-assisted telephone interview (CATI), the secondary mode (in case of CATI non-response) paper-and-pencil (up to panel wave 3) or CAWI (computer-assisted web interview, in later panel waves). The primary mode of the CQ is CAWI, with paper-and-pencil as secondary mode in case of CAWI non-response (see Hupka-Brunner et al., 2023 for more detail).

### 2.5 Survey instruments

Along with detailed student background characteristics, the baseline surveys of both TREE cohorts provide elaborate measurements of general literacy skills^8^ that are at the respondents’ command at the end of their compulsory schooling (9th grade). Documentation of these tests is under the responsibility of the OECD (in the case of TREE1’s baseline survey PISA 2000) and the Swiss Conference of Cantonal Ministers of Education (EDK, in the case of TREE2’s baseline survey AES). The respective documentations are provided along with the TREE data releases. It should be noted that the tests themselves must not be published for licensing and confidentiality reasons. The subsequent TREE panel waves then collect detailed data on education and labour market trajectories, which are contextualised by a rich set of complementary information on various life domains that have been identified in previous research as factors relevant for the respondents’ later transitions from education into working and adult life. Detailed documentation of survey instruments are provided for both cohorts and available when downloading the data from SWISSUbase (TREE, 2019, 2023).

### 2.6 Quality Control

TREE guarantees high standards in terms of quality control through all stages of the survey-data cycle. Field work is mandated to a professional survey institute^9^ and thoroughly supervised by the TREE survey management. The institute has a solid reputation for the implementation of large-scale, multi-language national scientific surveys in Switzerland. Survey instruments, documentation and interviewer training materials are designed and developed in close cooperation between the institute and TREE’s survey management. Ongoing quality control during field work is ensured by means of TREE staff being present in the survey institute’s CATI call centres, listening in and providing feedback on the conducted interviews. Furthermore, any adjustments of survey instruments, survey modes or field operation procedures are carefully tested on pretest samples prior to main field operation.

Before the TREE data are made available to the research community, they are subjected to thorough quality and consistency checks by the TREE data management (von Rotz et al., 2023).

### 2.7 Data anonymisation and ethical issues

When the TREE1 cohort was launched, approval by an ethics committee or review board was not required and not common practice in Switzerland. However, the study has, from its very beginning, explicitly adhered to research methods that strictly follow professional ethical guidelines and good scientific practice. Today, TREE data collection, treatment and publication complies strictly and formally with Swiss ethical and data protection legislation.

Students who participated in PISA 2000 had been asked their explicit consent to be contacted by TREE for a follow-up survey. Students were informed in advance about the purpose and scope of the TREE study and that participation was strictly voluntary. Data are thoroughly anonymised before publication.

Procedures of obtaining panel consent from the participants were largely the same in TREE2 as in TREE1. Due to more rigorous data protection legislation, TREE2 respondents were furthermore called to give their explicit consent that the data collected in the baseline survey (AES) may be linked with the subsequent panel data. A detailed data management plan guarantees strict confidentiality and security standards with regard to collection, treatment and transfer of contact and survey data.

### 2.8 Data use

TREE is funded as a social science data infrastructure by the Swiss National Science Foundation. Hence, the study’s very “raison d’être” is to provide scholars with empirical data to draw on for their analyses. Over the years, scholars from Switzerland and abroad have taken lively advantage of this opportunity. With a cumulative total of over 500 researchers who have so far downloaded the data from the SWISSUbase data archive, TREE belongs to the country’s most frequently used social scientific datasets.

Accordingly, the scientific output drawing on TREE data has reached a total of approximately 300 publications – and is continually increasing. The full list of publications, most of them available in full text, is available on the study website (see www.tree.unibe.ch/results).

## (3) Dataset description and access

The following section refers to the shared scientific use files (SUF) that are freely available to the scientific community and for academic teaching.

### 3.1 Repository location

Information on how to access the data, information on the SUFs and the latest data releases is provided at the SWISSUbase repository: www.swissubase.ch/de/catalogue/studies/12476/18017/overview

Furthermore, permanent DOI identifiers have been assigned:

TREE1: https://doi.org/10.23662/FORS-DS-816-7

TREE2: https://doi.org/10.48573/kz0d-8p12

### 3.2 Object/file name

The TREE data and their documentation are downloadable from the SWISSUbase data archive (see DOI reference numbers in Section 3.1; see also TREE, 2019, 2023). TREE1 data are extensively documented in German, French and English, TREE2 data in English only.

The contents (at single file level) of the most recent available TREE1 data release are listed in Table 6. The contents of the most recent available TREE2 data release are listed in Table 7.

**Table 6:** Overview of TREE1 data and their documentation. Release 2019 (https://doi.org/10.23662/FORS-DS-816-7).


DATA	DOCUMENTATION

PISA 2000	

PISA-TREE_2000_Version_20xx.sav	– PISA_2000_manual_original-variables.pdf – PISA_2000_Codebook_complementary_20xx.pdf – PISA_2000_Technical_Report_original-variables.pdf – PISA 2000 school & student questionnaires in German, French, Italian and English – Warning re PISA 2000 variables on parental education (PISA_2000_Bergman_etal_2010_Problems_educ_attainment_parents.pdf)

**TREE1, WAVE-SPECIFIC DATA 2001–2014**	

*Notation:* TREE_data_wave-x-20yy_version_20zz.sav	*Notation:* TREE_codebook_wave-x-20yy_version_20zz.pdf

*Datasets of first panel wave, 2001:* TREE_data_wave-1–2014_version_2016_german.sav TREE_data_wave-1–2014_version_2016_french.sav	*Codebook for data of first panel wave, 2001:* TREE_codebook_wave-9–2014_version_2016.pdf

*Datasets and codebooks of all further panel waves are organised and named the same way as for panel wave 1*.	

**TREE1, FURTHER DATA**	

TREE_data_certificates_2001–2014_version_20xx.sav	TREE_codebook_certificates_2001–2014_version_20xx.pdf

TREE_data_weights_wave1–9_version_20xx.sav	– TREE_codebook_weights_wave1–9_version_20xx.pdf – Sacchi_2011_TREE_longitudinal_weights_german.pdf

TREE_job_episodes_2003–2014_version_20xx.sav	TREE_codebook_job_episodes_2003–2014_version_20xx.pdf


**Table 7:** Overview of TREE2 data and their documentation. Release 2023 (https://doi.org/10.48573/kz0d-8p12).


SECTION/FILES OF THE DATA PACKAGE		FILE NAME

**0 TO START WITH – GENERAL INFORMATION**		

	Working with the TREE2 data release: How to get started. Update 2023	TREE2_How-to-get-started_v2.pdf

	TREE2 study design. Update 2023	TREE2_Study_Design_Update_2023.pdf

	Notes on weighting and variance estimation. Update 2023	TREE2_Readme_Weights_v2.pdf

	Longitudinal Weights for theTREE2 panel survey. Construction and application	TREE2_Documentation_Weights.pdf

	The TREE multi-cohort panel study. Theoretical framework	TREE_Theoretical_Framework_2022.pdf

**1 DATASETS**		

	Data from panel wave 0 (baseline) (Stata format)	TREE2_Data_Wave_0_v2.dta

	Data from panel wave 0 (baseline) (SPSS format)	TREE2_Data_Wave_0_v2.sav

	Data from panel wave 1 (Stata format)	TREE2_Data_Wave_1_v2.dta

	Data from panel wave 1 (SPSS format)	TREE2_Data_Wave_1_v2.sav

	Data from panel wave 2 (Stata format)	TREE2_Data_Wave_2_v2.dta

	Data from panel wave 2 (SPSS format)	TREE2_Data_Wave_2_v2.sav

	Data from panel wave 3 (Stata format)	TREE2_Data_Wave_3_v2.dta

	Data from panel wave 3 (SPSS format)	TREE2_Data_Wave_3_v2.sav

	Activity spells data (episodes) from panel waves 0 to 3 (Stata format)	TREE2_Data_Episodes_v2.dta

	Activity spells data (episodes) from panel waves 0 to 3 (SPSS format)	TREE2_Data_Episodes_v2.sav

	Weights for waves 0–3 [Stata format]	TREE2_Data_Survey_Weights_v2.dta

	Weights for waves 0–3 [SPSS format]	TREE2_Data_Survey_Weights_v2.sav

**1–1 MATHS TEST (WAVE 0)**		

	Additional test data from baseline survey (Stata format)	TREE2_Data_Wave_0_Maths_Test_v2.dta

	Additional test data from baseline survey (SPSS format)	TREE2_Data_Wave_0_Maths_Test_v2.sav

	Read-me document for additional test data	TREE2_Readme_Wave_0_Maths_Test_v2.pdf

**1–2 – SCHOOL, CLASS AND SURVEY CONTEXT (WAVE 0)**		

	Additional school and sampling data from baseline survey (Stata format)	TREE2_Data_Wave_0_Schooling_Context_v2.dta

	Additional school and sampling data from baseline survey (SPSS format)	TREE2_Data_Wave_0_Schooling_Context_v2.sav

	Read-me document for additional school and sampling data from baseline survey	TREE2_Readme_Wave_0_Schooling_Context_v2.pdf

**1–3 COGNITIVE ABILITY TEST CAT (WAVE 0)**		

	Cognitive ability test data (Stata format)	TREE2_Data_Wave_0_CAT_v2.dta

	Cognitive ability test data (SPSS format)	TREE2_Data_Wave_0_CAT_v2.sav

	Syntax for cognitive ability test data (Stata format)	TREE2_Syntax_Wave_0_CAT_Validation_v2.do

	Read-me/short documentation on cognitive ability test data	TREE2_Readme_Wave_0_CAT_v2.pdf

**1–4 READING SPEED TEST**		

	Reading speed test data: scores only, graduation year subsample [Stata format]	TREE2_Data_Reading_Speed_Test_Graduation_Year_v2.dta

	Reading speed test data: scores only graduation year subsample [SPSS format]	TREE2_Data_Reading_Speed_Test_Graduation_Year_v2.sav

	Reading speed test data: all test variables, all test cases [Stata format]	TREE2_Data_Reading_Speed_Test_Items_v2.dta

	Reading speed test data: all test variables, all test cases [SPSS format]	TREE2_Data_Reading_Speed_Test_Items_v2.sav

	Reading speed test data: syntax (Stata format)	TREE2_Syntax_Reading_Speed_Test_Validation_v2.do

**1–5 PRE-GRADUATION MEASUREMENTS**		

	Data on measures collected specifically prior to upper secondary graduation (Stata format)	TREE2_Data_Pregraduation_v2.dta

	Data on measures collected specifically prior to upper secondary graduation (SPSS format)	TREE2_Data_Pregraduation_v2.sav

	Read-me/short documentation on pre-graduation measurements	TREE2_Readme_Pregraduation_v2.pdf

**2 CODEBOOKS, VARIABLE LISTS & CODE LISTS**		

	Codebook for data of panel wave 0 (baseline survey, 2016)	TREE2_Codebook_Wave_0_v2.pdf

	Codebook for data of panel wave 1 (2017)	TREE2_Codebook_Wave_1_v2.pdf

	Codebook for data of panel wave 2 (2018)	TREE2_Codebook_Wave_2_v2.pdf

	Codebook for data of panel wave 3 (2019)	TREE2_Codebook_Wave_3_v2.pdf

	Codebook for episodic data	TREE2_Codebook_Episodes_v2.pdf

	Codebook for weighting data	TREE2_Codebook_Weights_v2.pdf

	Technical lists of variables, panel waves 0–3 (2016–2019)	TREE2_Variable_Lists_Technical_v2.pdf

	Conceptual list of variables, panel waves 0–3 (2016–2019)	TREE2_Variable_List_Conceptual_v2.xlsx

	Code list of occupations	TREE2_Codelist_Occupations_v2.xlsx

	Code list of education programmes	TREE2_Codelist_Education_v2.xlsx

**3 DOCUMENTATION ON SCALES**		

	Documentation of scales implemented from panel wave 1 onward	TREE2_Scale-Reporting_Wave_1_onwards_v2.pdf

**4 QUESTIONNAIRES**		

	Questionnaire administered in panel wave 0 (baseline survey, AES), German	TREE2_Questionnaire_Wave_0_AES_GER

	Questionnaire administered in panel wave 0 (baseline survey, AES), French	TREE2_Questionnaire_Wave_0_AES_FRE

	Questionnaire administered in panel wave 0 (baseline survey, AES), Italian	TREE2_Questionnaire_Wave_0_AES_ITA

	Questionnaire administered in panel wave 0 (baseline survey, AES extension), German	TREE2_Questionnaire_Wave_0_AES_Extension_GER

	Questionnaire administered in panel wave 0 (baseline survey, AES extension), French	TREE2_Questionnaire_Wave_0_AES_Extension_FRE

	Questionnaire administered in panel wave 0 (baseline survey, AES extension), Italian	TREE2_Questionnaire_Wave_0_AES_Extension_ITA

	Complementary questionnaire administered in panel wave 1, German	TREE2_Questionnaire_Complementary_Wave_1_GER

	Complementary questionnaire administered in panel wave 1, French	TREE2_Questionnaire_Complementary_Wave_1_FRE

	Complementary questionnaire administered in panel wave 1, Italian	TREE2_Questionnaire_Complementary_Wave_1_ITA

	Complementary questionnaire administered in panel wave 2, German	TREE2_Questionnaire_Complementary_Wave_2_GER

	Complementary questionnaire administered in panel wave 2, French	TREE2_Questionnaire_Complementary_Wave_2_FRE

	Complementary questionnaire administered in panel wave 2, Italian	TREE2_Questionnaire_Complementary_Wave_2_ITA

	Complementary questionnaire administered in panel wave 3, German	TREE2_Questionnaire_Complementary_Wave_3_GER

	Complementary questionnaire administered in panel wave 3, French	TREE2_Questionnaire_Complementary_Wave_3_FRE

	Complementary questionnaire administered in panel wave 3, Italian	TREE2_Questionnaire_Complementary_Wave_3_ITA


### 3.3 Data type

The TREE data are basically numerical (at all scale levels). Categorial and open text data are exhaustively numerically coded.

### 3.4 Format names and versions

The datasets are published in Stata and SPSS formats. Data documentation is provided in PDF and Excel formats. Versioning and releasing of the published data observes international standards and is supervised and implemented by the SWISSUbase repository at FORS center in Lausanne.^10^

### 3.5 Language

TREE is a multi-lingual study collecting data in several survey languages. Therefore, parts of the documentation and data are available in German, French and Italian. For TREE1, the most important documents (study design, concepts and scales, etc.) are provided in English, German, and French. For the second TREE cohort, documentation and labelling of numerical data is available in English only. However, data releases comprise exhaustive documentation of the questionnaires’ wordings in the three national survey languages (German, French and Italian).

### 3.6 License

The TREE data, as published in the SWISSUbase repository, are basically freely available for scientific use. However, given the sensitivity and comprehensiveness of the collected panel data on the surveyed individuals, there are minimal restrictions to the download of the data (see Section 3.7 for detail).

### 3.7 Limits to sharing

The TREE data comprise sensitive information. In compliance with data protection regulations, the datasets are available as scientific use files for scientific research and academic teaching only. Access to the data is therefore controlled by the data producers (i.e., TREE). This is custom procedure at the SWISSUbase repository for any sensitive individual data, for which data protection laws prevent open sharing. The data are made available to users upon prior (online) signature of a user agreement and submission of a short description of the type of (scientific) use that the signatories of the agreement plan.

Among other things, the user agreement specifies that

– the data may only be used for the declared research purpose
– the data must not be used for commercial purposes
– users must not attempt to identify individuals
– the data must be properly cited
– upon expiration of the agreement, the data and all related materials must be deleted (and deletion confirmed vis-à-vis the data provider).

Duration of the agreement’s validity is limited to a maximum of 24 months. Validity of the agreement may be extended at any time.

### 3.8 Publication date

The most recent TREE1 data release has been published in May 2019 (TREE, 2019).

The most recent TREE2 data release has been published in June 2023 (TREE, 2023).

### 3.9 FAIR data/Codebook

Data published in the SWISSUbase data repository – and hence TREE – fully conform to the FAIR principles. Findability is further enhanced by the fact that SWISSUbase datasets are listed in the CESSDA catalogue.^11^

## (4) Reuse potential

As a data infrastructure for the social sciences, reuse of TREE’s data is the very “raison d’être” of the study. The fact that to date, over 500 scholars have made analytic use of the TREE data is living proof of this.

Despite the large number of TREE-based analyses conducted so far, their analytical potential is far from being exhausted. Not least because of TREE’s multidisciplinary nature, the data have repeatedly led to analyses in a variety of disciplines, including sociology, economics, education, migration studies, psychology, and life course research in general. In contrast, interdisciplinary analyses have been rare, and hence present substantial untapped analytic potential. In addition, the panel surveys of both TREE cohorts are ongoing, with further panel waves planned in the medium- to long-term future. With the data collection of each additional panel wave, the reuse potential of the TREE data increases for at least two main reasons: On the one hand, the observation period of the panel is continually extended, and thus the respondents’ life span covered by the data. On the other hand, the same applies to the biographical period across which the two TREE cohorts can be analytically compared with each other. First analyses (Gomensoro & Meyer, 2021) show the great value of comparing the two TREE cohorts with regard to their educational pathways. The long (and continually growing) observation span of the data allows for the analysis of long-term developments and effects, e.g., cumulative (dis)advantages or (intergenerational) social mobility (see, e.g., Gomensoro & Bolzman, 2019). Furthermore, the multi-cohort design of the TREE study may yet be strengthened if, as planned, further school leavers’ cohorts are launched in the future.

TREE’s survey program covers various aspects of the life course such as educational and employment trajectories, intra-individual development, and different life domains (family and networks, leisure, health, social participation). While analyses of educational and employment trajectories are comparatively often at the center of previous analyses, analyses that consider intra-individual development or effects on several life domains have been scarce to date – and hence represent great, untapped reuse potential. In addition, the measurement of competencies (both at baseline and longitudinally) was improved for the second TREE cohort. Thus, the TREE2 data offer previously untapped analytic potential for improved analyses of how competencies influence different life domains and how competencies develop during post-compulsory education.

Owing to the careful alignment of survey instruments with similar panel surveys in other countries, the data also lend themselves to cross-national comparisons (see, e.g., Murdoch et al., 2017).

Reuse potential is further fostered by the fact that TREE lends itself for mixed-methods studies/analyses. A recent and promising example for this is the PICE (Parental Investment in Children’s Education, see www.pice.unibe.ch) add-on study conducted from 2021 to 2023: PICE collected qualitative data among approximately 70 TREE respondents and their parents, thus allowing to combine the analytic potential of both quantitative and qualitative data.

In a methodological perspective, the temporally fine-grained (month-by-month) and richly contextualised panel data on activity spells allow for different panel modelling techniques such as sequence analyses, event history analysis, structural equation modelling in general and latent growth or cross-lagged analyses in particular (see, e.g., Keller et al., 2019; Samuel et al., 2013; Tschopp et al., 2015) but also propensity score matching or fixed-effects models.

## Footnotes

For details on TREE’s theoretical framework see Hupka-Brunner et al. (2022).

Contrary to international standards, the Swiss education authorities include two years of kindergarten (i.e., preschool education) to a total of 11 years of compulsory schooling.

The main test domain of the PISA 2000 assessment was reading literacy, with mathematics and science literacy as secondary domains.

We provided a complete, long version and an abbreviated short version of the questionnaire administered by telephone. Throughout the four panel waves in question, 7 to 17 percent of all respondents made use of this mode (see TREE, 2016d).

I.e., interviewers draw on data collected in previous panel waves (for detail, see Rudin & Müller, 2013).

As of panel wave 10 (2019/20), we switched to CAWI as primary mode for the CQ.

See www.uegk-schweiz.ch for more detail.

For a general discussion of the literacy concepts adopted by large-scale assessments such as PISA, see OECD (2006; 2013; 2021; OECD & HRD Canada, 1997). The test design of the Swiss AES scheme draws on these concepts to a large extent.

TREE1, panel waves 5 to 7, LINK Institut, Lucerne. TREE1, panel wave 8 onward & TREE2; M.I.S. Trend, Lausanne.

https://www.swissubase.ch/en/catalogue/studies/12476/18017/overview.

Consortium of European Social Science Data Archives (see https://datacatalogue.cessda.eu/).
